# Activity Assessment of Antibiotics Used Against Different Bacterial Etiological Agents of UTI in Najaf, Iraq

**DOI:** 10.30699/ijp.2024.2027209.3293

**Published:** 2024-08-09

**Authors:** Mohammed Jasim Al-Shamarti

**Affiliations:** Department of Pathological Analysis, Faculty of Science, University of Kufa, Najaf, Iraq

**Keywords:** Antibiotic resistance, Pathogenic bacteria, Urinary tract infection

## Abstract

**Background & Objective:**

Antibiotic resistance in urinary tract infection (UTI) is increasing nowadays, therefore, the aim of this study was to evaluate the resistance patterns of many pathogens toward several antibiotics that are in common use in our hospitals.

**Methods:**

Subculture and identification of pathogenic bacteria were performed on 1148 hospitals' bacterial primary cultures which were considered positive for UTI. An antibiotic sensitivity test was performed by using the disc diffusion method. The rates of resistance were statistically analyzed and correlated with the types of antibiotics and bacteria.

**Results:**

It was found that 1148 out of 2087 urine samples were UTI positive, the majority of cases (76%) were from females (*P*<0.0001). *Escherichia coli* and *Klebsiella* were the most isolated Gram-negative bacteria, while Staphylococcus spp. was the most isolated Gram-positive pathogen. E. coli showed the highest resistance rate among all bacteria, while Streptococcus spp. was the most sensitive. The highest resistance was noticed to be against gentamicin and ampicillin, while the most effective drugs were imipenem and amikacin. There was a significant difference in resistance rates among the different bacterial categories (*P*<0.0001), while no significant difference was noticed in resistance rates among antibiotics categories (*P*>0.05).

**Conclusion:**

Elevated rates of antibiotic resistance were noticed in this study in UTI-causing bacteria; therefore, it is highly important at least to every general hospital to investigate the antibiotic resistance rates occasionally to determine the proper antimicrobial treatment as well as re-evaluate antibiotics which were considered as empirical.

## Introduction

Urinary tract infection (UTI) is considered one of the most commonly occurring infections among the human population with a higher incidence in females than in males (1-3). Several factors aid the pathogenic bacteria to invade the urinary tract and multiply to reach above 105 bacterial cells per milliliter in urine causing UTI (3). Serious damage to the urinary system usually occurs if the UTI-causing bacteria is left untreated (2, 4, 5). Antibiotics used randomly to treat UTIs lead to the emergence of antibiotic resistance and failure of UTI recovery as a result. The random use of antibiotics occurs when taking the treatment without relying on the findings of microbiology laboratories which diagnose the uropathogenic bacteria and their response to antibiotics (3, 6). Antibiotics helped humanity by treating serious infections. However, the antibiotic abundance and their increased use have led gradually to increased antibiotic resistance worldwide, mainly in developing countries (7, 8). The World Health Organization (WHO) has reported that antibiotic resistance is increasingly becoming a threat to modern medicine globally in 2022 (9). As antibiotics are being used improperly and prescribed incorrectly and unreasonably, resistant microbes are emerging continuously (10, 11). The proper antibiotic prescription must take into consideration the time, administration manner, and dose to meet rational and proper antimicrobial treatment (11, 12). Researchers have reported that 30-60% of the antibiotic treatments are prescribed randomly and therefore improperly without referring to the precise laboratory microbial diagnosis and antimicrobial sensitivity test (13, 14). Therefore, detecting the UTI-causing bacteria and their response to antibiotics in the laboratory makes the UTI treatment straightforward and avoids the emergence of resistant pathogens (2). 

The current study was conducted for the identification of bacterial pathogens related to UTI as well as evaluation of antibiotics activity in treating these pathogens in two main teaching hospitals (Al-Sadr and Al-Zahraa hospitals) in Najaf/ Iraq over one year (from November 2022 to December 2023). The percentage of resistant bacteria was calculated over the study period. The findings of this study should bring attention to the most effective antibiotics that might be used to treat UTIs, as well as warn the medical field about the increased rate of antibiotic resistance.

## Material and Methods

**Sample Collection and Study Design**

Samples included in this study were bacterial cultures (not urine) obtained from the microbiology laboratories in hospitals. Urine samples were cultured by the hospital medical laboratory team for patients who were clinically diagnosed to be suffering from UTI. We collected bacterial cultures that were previously isolated in hospitals and subcultures and further bacterial identification was performed for these bacterial samples. 

**Culture and Identification of Bacteria**

Subcultures from the hospital primary cultures were performed on selective media such as ECC ChromoSelect Selective Agar and MacConkey agar (Thermo Fisher, USA). Bacterial identification was conducted by biochemical tests such as citrate, catalase, indole, coagulase, methyl red, nitrate, H2S production, oxidase urease, and Voges Proskauer. all culture and biochemical tests were performed in accordance with microbiological manuals (17).

**Antibiotic Sensitivity Test**

According to the standards of the microbiology laboratory (18), the activity of several antibiotics was tested against bacteria on Mueller-Hinton agar by diffusing antibiotic discs. Various antibiotics were tested including Ampicillin, Amikacin, Cefalotin, Cefotaxime, Cefoxitin, Ceftriaxone, Ceftazidime, Ciprofloxacin, Imipenem, Gentamicin, Levofloxacin, Nitrofurantoin, and Norfloxacin which are available and commonly used routinely in our hospitals. The concentration of all antibiotics disks is 30 g from Thermo Fisher USA.

**Molecular Detection of CTX-M Beta-lactamase**

Plasmid DNA was isolated from bacteria using a Qiagen, USA plasmid isolation kit by following the manual leaflet inside the kit. DNA amplification by PCR was performed using specific primers for the *CTX-M* gene and Taq-polymerase master mix (Promega, USA). The forward and reverse primer sequences are 5'-CGCTTTGCGATGTGCAG-3' and 5'-ACCGCGATATCGTTGGT-3' respectively which are synthesized by Macrogen, South Korea. The thermal cycling (using a PCR thermal cycler from Bio-Rad, USA) of the PCR reaction was carried out in 35 cycles of three steps; denaturation (94°C for 30 seconds), annealing (60°C for 1 minute), and elongation (72°C for 1 minute) (19). Electrophoresis for the PCR products was performed using 1% agarose gel containing ethidium bromide (Thermo Fisher USA) and then visualized by UV illuminator (Bio-Rad, USA). 

**Statistical Analysis**

The percentages of resistance of each bacterium against each type of antibiotic were considered in the statistics. Non-parametric statistical tests (for not normally distributed data) in SPSS 26 (SPSS Inc., Chicago, Ill., USA) were used in this study. Mann-Whitney U test was used to analyze the incidence of UTI caused by different types of bacteria according to the patient's gender. Mann-Whitney U test was also used to analyze the significant difference in resistance rates between Gram-negative and Gram-positive bacteria. Kruskal-Wallis H test was used to test the significant difference of resistance among the different categories of both bacteria and antibiotics. A P-value≤0.05 was considered significant.

## Results

**Positive Cutlers of UTI and Bacterial Incidence**

The urine samples processed by the hospital medical team were 2087. Out of them, 1148 (55%) were positive for UTI. *Escherichia coli*, *Klebsiella spp*., and *Staphylococcus spp*. were the most prevalent isolated bacteria comprising 555 (48%), 178 (16%), and 172 (15%) respectively from the total number of positive UTIs.

Female patients constituted the majority (76%) in all cases of positive UTI which were distributed among different bacterial categories (*P*<0.0001) (Table 1).

**Antibiotic Resistance**

The resistance of each bacterium toward each antimicrobial agent was represented as a percentage (Table 2). The statistical analysis showed a strong significant difference in resistance among the different groups of bacteria (*P*<0.0001). Among all bacterial categories, *E. coli* and *Klebsiella* were the most resistant bacteria showing resistance rates of 82.4% and 82% respectively, while *Streptococcus* and *Staphylococcus* were the most sensitive showing resistance rates of 15% and 19% respectively. Gram-negative bacteria generally showed higher resistance than Gram-positive bacteria (*P*<0.0001).

The statistical test revealed no significant difference in resistance toward the different categories of antibiotics in this study (*P*>0.05). The results showed that Imipenem, Amikacin, and Ciprofloxacin, respectively, were the most effective antibiotics against all bacteria in the current study (Table 2). Bacterial standard strain *Klebsiella pneumoniae* ATCC700603 was used as the negative control.

**Table 1:** The frequency of isolated bacteria from positive UTI samples correlated with the patient's gender

**Bacteria**	**Male**	**Female**	**Total**	**Bacteria Percentage**	**Female%**	**Male%**
***E. coli***	98	457	555	48%	82%	18%
***Klebsiella spp.***	45	133	178	16%	75%	25%
***Staphylococcus spp.***	58	114	172	15%	66%	34%
***Enterococcus spp.***	19	43	62	5%	69%	31%
***Enterobacter spp.***	12	44	56	5%	79%	21%
***Streptococcus spp.***	20	31	51	4%	61%	39%
***Proteus spp.***	9	31	40	3%	78%	23%
***Pseudomonas spp.***	10	11	21	2%	52%	48%
**Others**	5	8	13	1%	62%	38%
**Total**	276	872	1148	100%	76%	24%

**Table 2:** The rate of antibiotic resistance expressed by different bacterial categories.

Bacteria	*E. coli*	*Klebsiella spp.*	*Staphylococcus spp.*	*Enterococcus spp.*	*Enterobacter spp.*	*Streptococcus spp.*	*Proteus spp.*	*Pseudomonas spp.*	Total
Antibiotic	Resistance percentage (%)								
Ampicillin	89.1	94	15	16	100	11	80	97	62.8125
Amikacin	43.1	54	5	81	48	12	15	20	34.725
Cefalotin	99.7	99	25	22	100	20	33	95	61.7
Cefotaxime	98.2	93	18	20	97	13	23	74	54.4875
Cefoxitin	93.2	87	20	24	95	15	30	91	56.9125
Ceftriaxone	98.6	100	41	46	99	16	22	70	61.5125
Ceftazidime	98.2	94	33	22	93	17	42	65	58.0125
Ciprofloxacin	54	44	24	61	40	25	18	33	37.4625
Imipenem	60	68	0	68	45	5	8	23	34.5375
Gentamicin	95.9	92	20	97	90	22	24	95	66.9875
Levofloxacin	70.3	76	19	71	63	18	16	30	45.4
Nitrofurantoin	73	80	13	89	60	12	100	60	60.825
Norfloxacin	98.4	88	17	90	97	10	17	37	56.8125
Total resistance (%)	82.4	82	19	54	79	15	32.9	61	53.24519

**CTX-M Gene PCR Amplification**

**Fig. 1:**
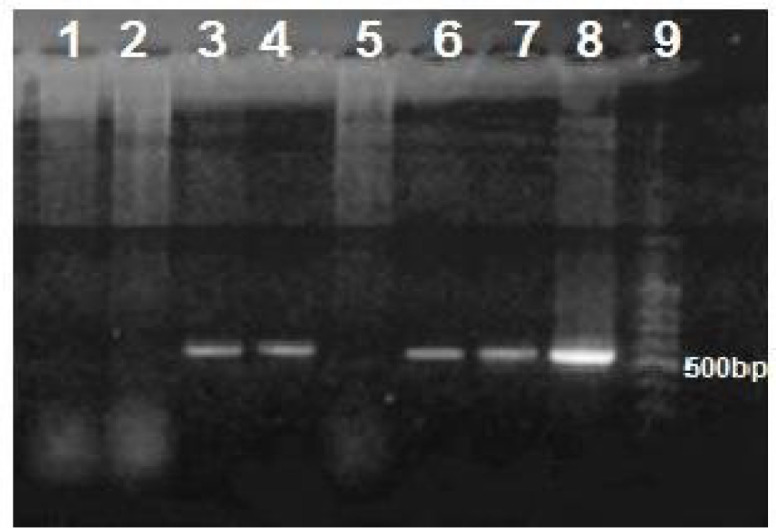
Agarose gel electrophoresis for PCR products of CTX-M gene amplification. Lanes 3, 4, 6, 7, and 8 are showing positive PCR amplifications that are 566 bp in size. Lanes 1, 2, and 5 are showing negative PCR amplifications. Lane 9 is a standard DNA ladder.

The molecular detection of CTX-M beta-lactamase was performed on 50 *E. coli* isolates that exhibited the highest rates of antibiotic resistance. The PCR amplification results revealed that 32 (64%) isolates were positive for the *CTX-M* gene (Figure 1). Bacterial standard strain *K. pneumoniae* ATCC700603 was used as the negative control.

## Discussion

In this study, we aimed to investigate the bacterial causative agents of UTI in our hospitals and to test the antimicrobial resistance toward isolated pathogens. In such a manner, it has been possible to detect the most isolated bacteria from UTI, the most resistant bacteria, as well as the most effective antibiotic. It has been found that gram-negative bacteria are more common in UTIs than gram-positive.

 We found that *E. coli* was the dominant bacteria to cause UTI comprising 48% of the total isolates. We found that cefalotin was the most antibiotic to be resisted by *E. coli* (resistance rate 99.7%), while the most effective antibiotic against *E. coli* was amikacin (resistance rate 43.1%). It has been reported by several studies that *E. coli* is the most common pathogen to be isolated from the urine of patients diagnosed with UTI (20-22).

The second most commonly isolated UTI pathogen was *Klebsiella spp.* which was found in 16% of UTI-positive samples. *Klebsiella spp.* exhibited high rates of antibiotic resistance, especially to ceftriaxone (resistance rate 100%). However, ciprofloxacin showed the highest activity toward *Klebsiella spp.* (resistance rate 44%). These results agreed with many studies that considered *Klebsiella spp.* as the second main Gram-negative *Enterobacter*iaceae pathogen of UTI (21-23).

We found that *Staphylococcus* spp. was the most prevalent Gram-positive bacteria isolated from UTI samples. This result is consistent with the findings of other studies (22, 24, 25).

The majority of UTI-positive samples (76%) were obtained from female patients, and this is due to the anatomical difference between male and female urethra which aids adherence and colonization of opportunistic bacteria (26, 27).

Recently, the use of antibiotics turned out to be improper due to their availability and widespread of them. This, however, made the UTI bacterial pathogens to be challenging to treat due to the increased rates of antimicrobial resistance worldwide (28). Furthermore, repeated UTI and urinary tract abnormality increase the chance of developing antibiotic resistance (29). Antibiotic treatment for UTI is usually given before or even without sending urine samples to the laboratory for testing the antimicrobial sensitivity. Initiating antibiotic treatment courses in such a manner is related to the emergence of resistance in pathogens due to the most likely wrong prescribed drug (30). 

The statistical analysis in the current study showed that there was no significant difference in resistance among different antibiotics. In other words, different antibiotics have been resisted approximately equally by different bacteria, while resistance rates were significantly different among bacterial categories, which means different bacteria showed different resistance rates. All bacteria appeared to have the highest resistance against gentamicin and ampicillin, while the least resistance was reported in imipenem and amikacin. The latter findings are consistent with a study carried out in the north of Iran (22). The most resistant bacterium to all antibiotics was *E. coli*, while *Streptococcus spp.* was the most sensitive.

Different antibiotic resistance patterns in pathogens of UTI were reported in different countries. In Iran, methicillin was reported as the most common antibiotic to be resisted by UTI bacteria, especially Staphylococcus (75%) (22). In Turkey, Ampicillin was found as the less effective drug, while meropenem is the most active in UTI treatment (21).

The ability of pathogens to spread and share antibiotic resistance has been reported to occur mainly among Gram-negative bacteria. Among Gram-negative, *Pseudomonas* and *E. coli* were found to be the most multidrug-resistant (MDR) bacteria especially when isolated from UTI (24, 31). In addition to the MDR, pan-drug resistance (PDR) has become a challenge in Acinetobacter isolated from UTI (22, 32, 33).

In this study, bacteria, especially Gram-negative, showed raised rates of resistance against antibiotics that contain beta-lactam ring in their structure due to bacteria's ability to produce beta-lactamase enzymes which destroy the antibiotic beta-lactam ring (34). Therefore, carbapenem antibiotics such as imipenem and meropenem, which lack beta-lactam rings in their structure, have become the drugs of choice to treat UTIs that express resistance to treatment by other antibiotics. However, resistance to carbapenems (imipenem) antibiotics was reported in the current study. Therefore, health-responsible authorities must be aware of developing antibiotic resistance that can make treatment of UTI and/or other bacterial infections a significant problem. 

We examined the presence of CTX-M as it represents the gene of the most prevalent beta-lactamase in multi-drug-resistant bacteria, especially *E. coli* (35-38). The most resistant *E. coli* isolates were considered as candidates for the molecular test in the current study. By such a test, we examined the ability of bacteria to develop genetic alteration for the sake of survival in the urinary tract where antibiotics are present as treatments. The *E. coli* isolates that exhibited beta-lactam antibiotic resistance with no CTX-M gene found in them explain the fact of having other types of beta-lactamase genes in these isolates. In another study, It has been found that 84.9% of *E. coli* isolates have either CTX-M or TEM beta-lactamases or both (39). It has been found that uropathogenic *E. coli* possesses the highest rate of extended-spectrum beta-lactamases expression (40). 

Studies that are concerned with the evaluation of antibiotic resistance rates in defined human populations are considered studies of significant importance. These studies should make the health authorities aware of infection treatment resistance. Antibiotic resistance studies must be performed in a parallel manner alongside the improper use of antibiotics in certain populations. Unmanaged and uncontrolled use of antibiotics triggers the accumulation of genetic alterations in pathogenic bacteria causing resistance to develop continuously over time and therefore resistance patterns to be different among pathogenic bacteria over time as well. The activity of non-beta lactam antibiotics such as imipenem, amikacin, and ciprofloxacin appeared to be higher against uropathogenic *E. coli*, therefore, such antibiotics can be used as a first-line choice in UTI treatment. 

## Conclusion

Using antibiotics without testing their activity against pathogens would most likely lead to improper use of treatment that triggers resistance by pathogenic bacteria. The results of the present study revealed increased rates of resistance. A significant difference was found in resistance among different UTI-causing bacteria, while no significant difference in resistance was found among different antibiotics. Molecular screening for the most prevalent beta-lactamase enzyme (CTX-M) in the most prevalent UTI etiological agent (E. coli) suggests a high incidence of genetic alteration and developing drug resistance in bacteria. Occasional screening and assessment of antibiotic resistance is highly recommended to evaluate the activity of empirical drugs prescribed against the etiological agents of UTI and other infectious bacterial diseases.
